# Promoting physical activity using an activity monitor and a tailored web-based advice: design of a randomized controlled trial [ISRCTN93896459]

**DOI:** 10.1186/1471-2458-5-134

**Published:** 2005-12-15

**Authors:** Sander M Slootmaker, Marijke JM Chin A Paw, Albertine J Schuit, Jacob C Seidell, Willem van Mechelen

**Affiliations:** 1 Institute for Research in Extramural Medicine, Department of Occupational and Public Health, Body@Work Research Centre Physical Activity, Work and Health, TNO-VU University Medical Centre, Amsterdam, The Netherlands; 2 National Institute for Public Health and the Environment, Bilthoven, The Netherlands; 3 Institute of Health Science, Faculty of Earth and Life Sciences, VU University Amsterdam, The Netherlands

## Abstract

**Background:**

Ageing is associated with a decrease in physical activity. This decrease particularly occurs during specific transitional life stages. Especially during adolescence and young adulthood a steep decrease in physical activity is observed. Inactive people are often not aware of their inactivity. Providing feedback on the actual physical activity level by an activity monitor can increase awareness and may in combination with an individually tailored physical activity advice stimulate a physically active lifestyle.

**Methods:**

In a randomized controlled trial the effectiveness of providing an activity monitor in combination with a personal physical activity advice through the Internet will be examined. Outcome measures are level of physical activity, determinants of physical activity, quality of life, empowerment, aerobic fitness and body composition. Participants are relatively inactive adolescents and young adults who are measured at baseline, after 3 months intervention and 5 months after the end of the intervention. In addition, facilitating and hindering factors for implementation of the intervention will be investigated.

**Discussion:**

The use of a personal activity monitor in combination with web-based assisted individually tailored health promotion offers a good opportunity to work interactively with large groups of adolescents and young adults and provide them with advice based on their actual activity level. It has great potential to motivate people to change their behaviour and to our knowledge has not been evaluated before.

## Background

### Realization of the research project

In 2002, PAM B.V. (Doorwerth, The Netherlands) introduced a commercially available innovative product and concept to promote physical activity. This concept consists of a physical activity monitor (PAM) as well as a specially designed website (PAM COACH) which gives personalized physical activity advice, based on the amount of physical activity measured by the physical activity monitor. After consultation with PAM B.V. (dr. E.P.N. Damen), the VU University Medical Centre (VUmc) and the National Institute for Public Health and the Environment decided to evaluate this concept by scientific research. An application for funding was made and granted by The Netherlands Organization for Health Research and Development (ZonMw), a non-profit organization. In order to conduct the PAM project, PAM B.V. sold the required PAMs to VUmc and facilitated access to the PAM COACH website, and provided us with technical PAM support. PAM B.V. was not a partner in the research proposal and in the further execution and processing of the study. The PAM project started in 2003 and will end mid-2006.

### Arguments for publishing the design of a study protocol

This article describes the design of a randomized controlled trial (RCT). Although the RCT has started already, no results are known at this point. Publishing the study design before the results are available has some important advantages. It offers the possibility to elaborate on the content of the intervention. This broad information gives users of similar interventions insight in the practicalities of the intervention and may promote wider implementation of the intervention if proven effective. Furthermore, it offers the opportunity to consider the methodological quality of the study more objectively, irrespective of the results.

### Introduction

The positive health benefits of physical activity in people of all ages have been extensively studied and are now commonly accepted. There is compelling evidence that regular physical activity increases physical functioning, aerobic fitness and quality of life and favourably influences many risk factors for chronic diseases, such as coronary heart disease, colon cancer and type 2 diabetes mellitus [1,2]. Nonetheless, people in industrialized countries continue to reduce their energy expenditure in daily living and at work. Leisure time activities have become more sedentary, with television watching, playing videogames, and personal computer usage among the most popular sedentary pastimes [3,4]. Especially during adolescence and young adulthood a steep decrease in physical activity is seen [5-7]. Adolescents are recommended to perform at least moderate physical activity with a minimum of one hour a day [8,9]. In 2004, only 31% of the male and 18% of the female adolescents (12–17 yrs) in the Netherlands complied with this guideline, according to data of the StatLine of the Dutch Central Bureau of Statistics [10]. Adults are advised to perform at least moderate physical activity for a minimum of 30 minutes on at least five days of the week. In 2004, only 50% of the Dutch young men and 55% of the women (25–35 yrs) were sufficiently active according to this guideline. An effective intervention to promote physical activity in these specific age-groups could therefore provide enormous health gains [11]. In a cross sectional study [12] among 2600 adults in the Netherlands was shown that 60% of the people that did not meet 30 minutes of physical activity on at least five days of the week, overestimated their own physical activity levels. Hence, feedback on the actual amount of physical activity per day may overcome this problem of poor self-evaluation and thereby positively affect the physical activity level among inactive persons.

One way to provide direct feedback on the actual amount of physical activity is via a physical activity monitor. Physical activity monitors can be worn without major inconvenience, requires little effort of the user and are compatible with most daily activities, making it a practical and socially acceptable measure of physical activity. Moreover, in a study of Rooney et al. [13], an increase in awareness of daily activity and increased physical activity was found, after wearing a pedometer for eight weeks.

Another way of providing feedback is via the computer. Computer-tailored interventions showed to be an acceptable, feasible and effective tool for promoting physical activity [14,15]. Furthermore, in a study of Brug et al. [16] computer tailored information appeared to have a greater impact in motivating people to change their diet than a general advice. The computer tailored information was more likely to be read, remembered and experienced as personally relevant compared to standard materials.

Internet websites enable to deliver tailored physical activity information on participants' desired moments and have the capability to easily reach large numbers of participants. Therefore, web based tailored interventions for physical activity could have great potential. However, until this point there is little evidence about the effectiveness of web-based-tailored interventions on physical activity. In an eight-week pilot intervention study of McKay [17], the intervention group received personalized feedback, received and could post messages to an on-line "personal coach", and were invited to participate in peer group support areas. This study found an overall moderate improvement in PA levels within both intervention and control conditions, but no significant between-group difference.

In our study we hypothesize that the use of a personal activity monitor (PAM) combined with an individually tailored physical activity advice will increase awareness and subsequent physical activity among inactive people. PAM-users will receive personal feedback and a personal physical activity advice. The use of an activity monitor in combination with a personal physical activity advice through the Internet is an innovative intervention that has not been evaluated before. The intervention is assumed to be attractive for adolescents and young adults. To date 84% of Dutch adolescents (12–17 yrs) and 71% of young adults (25–34 yrs) make use of the internet on a computer at home [18]. This underlines that the current generation of young people is accustomed to using computers and the Internet, and have the skills to participate in this intervention. In this randomized controlled trial the effectiveness of the PAM-accelerometer (PAM. B.V.) in combination with an individually tailored physical activity advice is evaluated in relatively inactive adolescents (12–18 yrs) and young adults (age 25–35 yrs).

## Methods

### Study design

**Figure 1:**
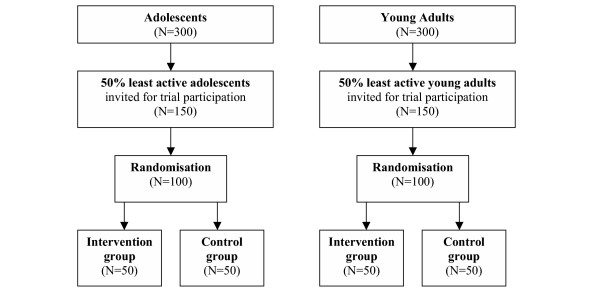
Participant flow chart.

The physical activity level of 300 adolescents and 300 young adults is monitored for two weeks. Based on these two monitor weeks, fifty percent relatively inactive adolescents and young adults are selected for the intervention study. In a randomized controlled trial (RCT) the effectiveness of a distributed PAM activity monitor coupled to a tailored physical activity advice through the Internet among adolescents and young adults in stimulating physical activity will be evaluated Effects on determinants of physical activity, quality of life, empowerment, aerobic fitness and body composition will be examined as well. In addition, a process evaluation is performed. See figure 1 for the flow chart of the participants.

### The PAM-concept

The Personal Activity Monitor (PAM, model AM101, 28 gr, 59 × 43 × 10 mm, PAM B.V.), is a uni-axial accelerometer in the vertical direction and comparable to the accelerometer developed by Meijer et al. [19]. The PAM can be attached easily to a belt and is worn on the right hip. The PAM produces a cumulative score, which is a proxy measure of total daily (24 h) physical activity. The PAM score is displayed continuously on the PAM. Via a docking station, which must be connected to a computer with an Internet connection, the user can upload his personal PAM scores through PAM software to the COACH database any time of the day. The COACH database is part of the COACH website. At first login on the COACH website, the users register themselves by filling out a form with personal data (i.e. height, weight) and a onetime brief questionnaire with 10 yes-no questions (e.g. Do you think you are able to walk to your work when it is raining?). Entering the COACH website, the user is asked to formulate an activity goal along with the COACH, based on the users' PAM score and age. On every subsequent login the COACH website presents all the collected PAM scores in orderly graphs per day which is supported by practical information on user preferred activities (e.g. daily an extra 60 minutes walking, or 25 minutes running, or 20 minutes playing squash). In addition, the user receives on subsequent logins tailored feedback on determinants of physical activity based on the answers of the onetime questionnaire at registration. Next to the feedback on the COACH website, the users can easily monitor their progress in daily physical activity by reading their score on the display of the PAM itself. Users can thus compare their progress with their set personal activity goal. Furthermore some features are changed with the cooperation of Pam B.V at the original COACH website in aid of the intervention. Unlike the original COACH website, the intervention COACH website will neither formulate a weight goal, nor give nutritional advice. Furthermore, general practical advices to implement physical activity in daily life are added to the site. A different advice is given on each subsequent login.

### Study population

The study includes apparently healthy adolescents, aged 12 to 18 years old and young adults, aged 25 to 35 years old. Inclusion criteria are ability to walk, speak Dutch and not being pregnant. The study protocol is approved by the Medical Ethics Committee of VU University Medical Centre before the start of data collection. The adolescents will be instructed to take the consent materials home to their parents, read the materials with their parents, and return the consent, signed by both the parent and the adolescent if they are willing to participate in the study. Informed consent will also be requested from the young adults.

### Recruitment

#### Adolescents

Adolescents will be recruited from high schools in the surroundings of Amsterdam. Enrolling students at a school site has the following benefits:

1. All ages in the age-range from 12 to 18 years are represented;

2. Students have fixed classes according to a fixed time schedule which facilitate the measurements;

3. Different educational levels can be selected;

4. At school students have access to computers with internet at school;

Schools in the surroundings of Amsterdam with a minimum of 1000 students and with different educational levels are approached by phone. In all schools first the physical education (PE) teacher are contacted. If the PE staff is interested, an appointment is made for a personal interview. The PE teachers are asked to assist with the recruitment and facilitation of the outcome measurements. After approval of the school board and the PE section, the project is presented to the children during the PE-class by the study staff and the adolescents receive an information leaflet with consent materials. The adolescent and his parents had one week to hand in the informed consent forms, which are collected at the day of the first measurement for the selection of the intervention. At each school one regular school day in the week is selected to reach different ages and educational levels.

#### Young adults

Young adults are recruited from worksites in the surrounding of Amsterdam. It was decided to include office workers, since they perform mostly sedentary work and are constrained to their work place. This simplifies recruitment of the participants and measurements at the worksite. Companies with mainly office workers are selected, and either their occupational health and safety coordinator or an employee of human resources is approached by phone. If the company is interested, a face-to-face meeting is arranged. If all stakeholders are informed and have given their approval, information on the project is distributed to the employees according to the companies' policy. The company is asked for assistance with the recruitment and to facilitate the measurements. Every employee receives identical information about the project; i.e. a recommendation letter of the occupational health and safety coordinator and an information brochure with a consent form. The consents are collected at the day of the first measurement. The measurements of the selection for the intervention as well as the measurements of the intervention take place during working hours at the worksite.

### Selection for intervention

**Figure 2:**
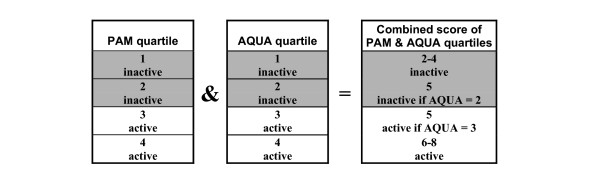
Combined physical activity score based on the activity quartiles of the PAM and AQUA.

The target group for the intervention study is relatively inactive subjects. In order to select these subjects, 300 adolescents and 300 young adults are asked to wear a PAM accelerometer during their daily activities for two weeks. The PAM display is blinded during this period. After this two weeks-period all participants are asked to fill in a short physical activity questionnaire (an adapted version of the SQUASH questionnaire; [20]). The adolescents and young adults are divided in an active and inactive group by means of a combined physical activity score. Physical activity data of the PAM and the AQUA are divided in four quartiles, stratified for educational level. The first quartile includes the most inactive subjects. For each subject the quartile-numbers of both the PAM and AQUA will be summed up. Those who are classified as 'inactive' (combined score lower than 5) will be invited for participation in the intervention study. When the combined score is five, the AQUA quartile plays a decisive role (See figure 2)

### Intervention

The subjects classified as relatively inactive will be invited to take part in the intervention study. For the intervention study they will be required to fill in a questionnaire and to participate in physical measurements (i.e. baseline measurements). After completion of the baseline measurements the participant are randomly assigned to the intervention or the control group. Participants in the intervention group receive the PAM monitor and have access to their personal website (COACH) for a 3-month period. The participants are given written and verbal instructions and practical demonstrations on how to wear the PAM and how to use the COACH. Special attention is paid to:

1. placing the PAM on the hip;

2. connecting the docking station to the computer;

3. installing the software on the computer;

4. registering and logon to the PAM COACH website;

5. setting a PAM goal and reading the results at the COACH website.

The control group receives a single written information brochure with general recommendations and guidelines for physical activity. Telephone numbers and an e-mail-address are provided in case questions arise. At each intervention site at least one computer with PAM software is arranged.

### Measurements

The primary outcome of the intervention study is the level of physical activity measured by questionnaire. Secondary outcomes are determinants of PA, quality of life, empowerment, aerobic fitness and body composition. A compact questionnaire is constructed to maximize the response and minimize time spent filling in the questionnaire. All measurements are performed at baseline and after 3 months of intervention. Five months after the 3 months intervention only the questionnaire is repeated.

### Questionnaire

#### Physical activity

The Activity Questionnaire for Adolescents & Adults (AQUA) is a short questionnaire developed to assess physical activity and sedentary activity at work, at school, during leisure time and transportation. The structure of the AQUA is obtained from the SQUASH-questionnaire [20]. Information on light (range for adolescents, 2–5 MET and adults 2–4 MET), moderate (range for adolescents, 5–8 MET and adults 4–6.5 MET) and vigorous (adolescents, >8 MET and adults >6.5 MET) intensity physical activities are obtained, as well as information on sedentary behaviour (<2 MET), such as TV viewing and computer usage. The AQUA refers to activities in the past week (7-day recall). Physical activity will be expressed in minutes per week and in a total activity score. The total activity score will be calculated by multiplying the minutes per week by the actual MET score of the specific activity (MET/min/week).

#### Determinants of physical activity

In this questionnaire, the enquired determinants of physical activity refer to three categories of activity: sport, transportation and sedentary activities. To minimize time spent filling in the questionnaire for each determinant of physical activity a selection of two or three relevant questions is made. The following determinants of physical activity are assessed in both age groups:

***Behavioural intention ***is the cognitive representation of a person's readiness to perform physically active behaviour, and it is considered to be the immediate antecedent of behaviour. Behavioural intention was measured with one item (i.e. 'Do you intend to play sport more in the following three months?' with response categories ranging from 1 = 'no, certainly don't' to 5 = 'yes certainly do'). According to the Theory of Planned Behaviour [21], behaviour in general is determined primarily by behavioural intention and postulates that this intention is determined by three constructs: attitude, subjective norms and perceived behavioural control.

***Attitude***, the beliefs that are associated with physical activity behaviour and the evaluations of these beliefs. The questionnaire contains two questions about attitude (i.e. 'I like playing sports?' with response categories ranging from 1 = 'strongly disagree' to 5 = 'strongly agree').

***Social influences ***[22] such as social norms, social expectations, modelling and imitating. Social norms are determined by the normative beliefs of significant others about physical activity behaviour and the individual motivation to comply with these persons. The questionnaire of the young adults contains five questions about social influences [23], the questionnaire of the adolescents contains four questions regarding social influences (i.e. 'My friends think I should play sports' with response categories ranging from 1 = 'no, certainly don't' to 5 = 'yes certainly do'). For the categories sport and transportation, two questions about modelling are added.

***Self-efficacy expectations***, which are beliefs of a person about his abilities to perform physical activity. Increased self-efficacy will result in improved performance of this behaviour. The questionnaire contains two questions about self-efficacy [24] (i.e. 'Do you think you can manage to play sports for at least half an hour a day?' with response categories ranging from 1 = 'no, certainly don't' to 5 = 'yes certainly do').

***Knowledge of daily physical activity ***is asked by one item (i.e. 'How much physical activity do you have to spend per day to stay healthy'?) with the response categories: one hour per week, three hours per week, thirty minutes per day, one hour per day, two hours per day no and don't know).

For the categories sport and transportation two extra determinants are assessed:

***Personal barriers***, many personal variables, including physiological, behavioural, and psychological factors, may affect our plans to become more physically active. Three items about personal barriers are asked [24] (i.e. 'Do you think you can manage to play sports when it rains?' with response categories ranging from 1 = 'no, certainly don't' to 5 = 'yes certainly do').

***Awareness of physical activity***, one item is included in the questionnaire (i.e. 'Do you think you spend enough time playing sports?' with the response categories yes and no). Awareness of the actual level of activity will be assessed by comparing the outcomes of the awareness questions with the total minutes per week of the concerning activity (i.e. sport, walking/cycling) from the questionnaire.

#### Quality of life

The quality of life of the adolescents is assessed by the Dutch version of the KIDSCREEN-10 Index [25]. The KIDSCREEN is a health related quality of life-measure (HRQOL) and applicable for healthy children aged from 8 to 18 years. The KIDSCREEN measures 10 HRQOL dimensions (Physical-, Psychological Well-being, Moods and Emotions, Self-Perception, Autonomy, Parent Relations and Home Life, Peers and Social Support, School Environment, Bullying, Financial Resources) by 11 items on a five-point scale, ranging from 1 "completely not" to 5 "completely". From the total score t-values and percentages will be calculated by age and gender.

The quality of life of the young adults is assessed by the SF-36 Health Survey (SF-36) [26,27]. The SF-36 provides a health related quality of life measure and is applicable for adults. The SF-36 is composed of 36 questions and standardized response choices, organized into eight multi-item scales: physical functioning, role limitations due to physical health problems, bodily pain, general health perceptions, vitality, social functioning, role limitations due to emotional problems and general mental health. All raw scale scores will be linearly converted to a 0 to 100 scale, with higher scores indicating higher levels of functioning or well-being. Item score and subscale scores will be calculated.

#### Empowerment

In both age-groups, empowerment is assessed with a Dutch version of the Pearlin Mastery Scale [28]. This scale refers to the extent to which a person has the feeling of being in control of his or her own life. The questionnaire consists of seven statements, such as 'Some of my problems I can't seem to be able to solve at all', with response categories ranging from 1 = 'strongly disagree' to 7 = 'strongly agree'. Higher scores on the scale indicate a greater sense of mastery. A total score will be calculated.

#### Process evaluation

Among the participants of the intervention group experience with the use of the activity monitor and the personal COACH will be evaluated. This will be assessed with a brief questionnaire with both qualitative and quantitative questions about the usage of the PAM and the PAM COACH website and possible problems that may have been experienced. Reasons for complying or not complying will be collected also, in order to obtain insight into the potential success or failure of implementation and long-term adherence user implementation.

### Physical measures

#### Aerobic fitness

In adolescents, the aerobic fitness is assessed by the 20 meter shuttle-run test (20 mSRT) [31,32]. The 20 mSRT is a maximal aerobic running test. The test starts at a speed of 8.0 km/h, which increases each minute by 0.5 km/h. To perform the test, the subject runs a 20-meter course back and forth. The pace is set by an audio signal. The test has been shown to be reliable and valid field test to estimate maximal aerobic capacity [31,33]. Changes in 20 mSRT score will be used as a measure for changes in aerobic capacity. The 20 mSRT is chosen for adolescents because the test can easily be carried out during the PE classes and most of the students are acquainted with the test.

In young adults, the aerobic fitness is assessed by the Chester Step Test (CST). Previous to every test each employee is screened with the Physical Activity Readiness Questionnaire (PAR-Q [34]). The PAR-Q will be interpreted by qualified staff. The CST is a submaximal test to predict aerobic capacity. The CST consists of increasing paces of stepping on and off a bench (on, on, off, off). The height of the bench is determined based on the age and self reported physical activity in the past week of the employee. The CST commences at the relatively slow pace of 15 steps per minute and increases every two minutes to 20, 25, 30 and 35 steps per minute. The stepping beat is set by a metronome (Yamaha Corporation, QT 1B). Throughout the test the heart rate is monitored continuously with a Polar (S610i) heart rate monitor (Polar, Electro Oy, Kempele, Finland). After each stage the subject will be asked to rate his perceived exertion on the Borg scale [35] which is a 15-point numerical rating scale, ranging from 6 "very very light" to 20 "exhaustion". The test is terminated when the subjects' heart rate reaches 80 percent of the age predicted maximal heart rate (i.e. 220 minus age), or when the subject rates 14 on the Borg scale. Aerobic capacity is predicted by the provided Chester Step Test calculator (ASSIST creative resources Limited, Wrexham, England). Changes in the CST score will be used as a measure for changes in aerobic capacity [36,37]. The CST is chosen for employees because the test is easy to perform at the worksite and it takes little time of the participants' time.

#### Body composition

Weight is measured in light clothing without shoes to the nearest 0.2 kg using a digital balance (SECA 888) and height is measured to the nearest 0.1 cm with a stadiometer (SECA 225). BMI (kg/m^2^) will be used as an index of relative weight. Waist-circumference was measured at the level of the belly button and the hip-circumference at the level of the trochanter-major. Both circumferences were measured using a spring loaded measuring tape (SECA 200) to the nearest 0.1 cm. The four skinfolds (biceps, triceps, subscapular and supra-iliac) are measured twice with a Harpenden skinfold caliper (Baty International, England) and averaged, according to earlier research for adolescents [29] and young adults [30]. If the difference between these two measurements was more than 1 mm, the measurement is done a third time.

### Sample size

The aim of the study is to establish a difference between the intervention and the control group of 20% in the proportion of subjects who are active according to the Dutch guidelines. Significance between the groups can be established with 80% (1-β) probability and a significance level of 0.05 (α). To be able to detect a difference of 20% in PA level, two groups of 50 subjects for each age group are required. Since we expect a dropout rate of 33 %, approximately 150 adolescents and 150 employees will be invited for the intervention.

### Statistical analysis

The primary analysis will be a comparison of the change in physical activity (PA) between the intervention and control group following the "intention to treat" principle. To adjust for the dependency between observations of subjects from the same school and class or company, multilevel analysis will be performed. Intention to treat analyses will also be performed for the secondary outcomes; determinants of physical activity, quality of life, empowerment, aerobic fitness and body composition. Pre-planned subgroup analyses will be performed for adolescents and adults and separate for gender. Where appropriate, adjustments will be made for differences at baseline values. Per protocol analyses will also be carried out.

## Discussion

The use of a personal activity monitor in combination with computer-directed health promotion offers a good opportunity to work interactively with large groups. This intervention method includes feedback on actual behaviour and is tailored to the individual. Since the advice given is developed interactively and takes preferences of the user into account, it is expected that physical activity can be included more easily in daily life. Additional advantages for the user of this individualized intervention are the direct feedback on the display and the flexibility of the personal COACH website system, i.e. the participants can decide when to login and monitor their stepwise progression in physical activity according to feedback information. The results of the intervention and process evaluation will contribute to the development of an effective preventive intervention and underpin the consideration whether introduction of such an intervention should be conducted at a larger scale, in the context of efficient and effective health care.

## Competing interests

None of the authors are involved in Pam B.V. The authors declare that they have no competing interests.

## Authors' contributions

MCAP and AJS designed the study, participated in the coordination of the study and in writing the article. WvM and JCS have been involved in the design of the study and in drafting the manuscript. SMS conducts the research and wrote the article. All authors provided comments on the drafts and have read and approved the final version.

## Pre-publication history

The pre-publication history for this paper can be accessed here:


